# Molecular Dynamics Simulation of Cracking Process of Bisphenol F Epoxy Resin under High-Energy Particle Impact

**DOI:** 10.3390/polym13244339

**Published:** 2021-12-11

**Authors:** Yunqi Xing, Yuanyuan Chen, Jiakai Chi, Jingquan Zheng, Wenbo Zhu, Xiaoxue Wang

**Affiliations:** 1 State Key Laboratory of Reliability and Intelligence of Electrical Equipment, Hebei University of Technology, Tianjin 300401, China; yqxing@hebut.edu.cn (Y.X.); yychebut@163.com (Y.C.); jiakaichi@gmail.com (J.C.); jqzhebut@163.com (J.Z.); 2 Key Laboratory of Electromagnetic Field and Electrical Apparatus Reliability of Hebei Province, Hebei University of Technology, Tianjin 300401, China; 3 China Southern Power Grid Research Institute Co., Ltd., Guangzhou 510080, China; zhuwb@csg.cn

**Keywords:** epoxy resin, partial discharge, active product, electro-thermal dissociation, reactive molecular dynamics

## Abstract

The current lead insulation of high-temperature superconductivity equipment is under the combined action of large temperature gradient field and strong electric field. Compared with a uniform temperature field, its electric field distortion is more serious, and it is easy to induce surface discharge to generate high-energy particles, destroy the insulation surface structure and accelerate insulation degradation. In this paper, the degradation reaction process of bisphenol F epoxy resin under the impact of high-energy particles, such as *O*_3_*^−^*, *HO^–^*, *H*_3_*O^+^* and *NO^+^*, is calculated based on ReaxFF simulation. According to the different types of high-energy particles under different voltage polarities, the micro-degradation mechanism, pyrolysis degree and pyrolysis products of epoxy resin are analyzed. The results show that in addition to the chemical reaction of high-energy particles with epoxy resin, their kinetic energy will also destroy the molecular structure of the material, causing the cross-linked epoxy resin to pyrolyze, and the impact of positive particles has a more obvious impact on the pyrolysis of epoxy resin.

## 1. Introduction

Epoxy resin is currently the polymer thermosetting insulating material with the highest share in industrial applications [1,2,3]. Among them, the bisphenol F epoxy resin is widely used in the insulation of extreme environment equipment, such as aerospace, superconductivity, medical and military industries due to its good low temperature performance [4,5]. For example, in the terminal of superconducting power equipment, the current lead is insulated with epoxy resin under the coupling action of a strong electric field and a large temperature gradient field, which easily induces partial discharge and accelerates insulation aging. Previous studies have shown that partial discharge (PD) is the main cause of insulation deterioration breakdown. The impact, corrosion and thermal effects of high-energy particles and active products produced by PD on the surface of insulating material will greatly influence the transient dielectric strength and aging process of insulating material [6,7,8].

At present, the research on insulation properties of polymer materials such as epoxy resins is mostly based on experiments. For example, there have been many studies on the extraction of characteristic parameters of partial discharge signal and the characterization methods [9,10,11] between partial discharge signal and material [12,13,14] insulation state and degradation process, and the aging cracking mechanism is analyzed based on the experimental data. However, it is difficult to reveal the aging micro-mechanism and degradation cracking process of insulation materials under partial discharge (PD) by experimental research, physical modeling and other methods. Furthermore, the research on simulation analysis at atomic level is mostly limited to the thermal decomposition process of macromolecule materials [15,16]. Few studies have been conducted on the influence mechanism of high-energy particle impact on aging and cracking of epoxy resin materials at atomic level.

ReaxFF force field uses quantum chemistry theory to judge the formation and breakage of chemical bonds and uses classical molecular dynamics force field to simulate the molecular conformation of the system. Combining the two effectively, ReaxFF force field can be widely used to simulate pyrolysis, detonation, particle impact, chemical reaction and other phenomena of various materials. By simulating the heating and cracking process of epoxy resin under microwave heating and conventional heating, Zhang Yiming reveals the cause of thermal runaway of macromolecular polymer system caused by microwave heating from a micro level [17]. Farzin Rahmani studied the mass loss, surface damage and penetration depth of AO in polyimide materials with different grafting methods bombarded by Atomic Oxygen (AO) based on ReaxFF force field [18]. Morrissey verified the feasibility of using ReaxFF force field to study the corrosion resistance of spacecraft metal materials under the impact of high-energy particles [19]. The above research results show that ReaxFF force field can be used to simulate the aging process of epoxy resin materials under the impact of partial discharge particles.

Under the action of strong electric field, air (main effective components are *N*_2_, *O*_2_, *H*_2_*O*) collides with electrons emitted by electrodes and positive and negative ions generated by ionization during partial discharge, resulting in particles with high energy and active products [7]. The high-energy particles generated during the discharge process have high kinetic energy under the action of strong electric field, and they continuously impact the epoxy resin surface. When impact occurs, the kinetic energy will be converted into internal energy, which will make the local temperature of insulation material rise sharply. At the same time, high-energy particles themselves will also have complex chemical reaction with insulation material, destroying the cross-linking structure of epoxy resin and accelerating the aging process of insulation. Chen X and Pavlik M carried out corona discharge studies on air under different humidity conditions and analyzed the species and activities of its products. The results showed that the highest reactivity and content was *O*_3_*^−^*, followed by *HO^−^* free radicals [20,21]. Wayne Sieck studied the corona discharge products of humid air and found that more than ten ion products with strong reaction activity were produced at the positive electrode, among which *H*_3_*O^+^* and *O*_2_*^+^* had the strongest reaction activity [22]. Y. Ehara et al. studied the degradation process of epoxy resin surface under partial discharge in different humidity air environments. The experiments proved that partial discharge can produce ozone, active oxygen atoms, nitrogen oxide, nitric acid and other active products [23]. Therefore, taking the above ion products as the object, it is of great significance to study the effect of high-energy particle impact produced by partial discharge on the cracking process of epoxy resin materials, so as to reveal its deterioration mechanism from the micro level.

In this paper, the interfacial model of cross-linked bisphenol F type epoxy resin and the models of hydration hydrogen ion (*H*_3_*O^+^*), nitric oxide ion (*NO^+^*), hydroxide ion (*HO^−^*) and ozone ion (*O*_3_*^−^*) were established. ReaxFF force field was used to inject the above four particles into the epoxy resin interface. The mechanism of high-energy particle impact caused by partial discharge on the aging and cracking process of epoxy resin and its reaction products were analyzed. The micro-aging mechanism of epoxy insulation material under partial discharge was revealed at atomic level.

## 2. Molecular Simulation Design

### 2.1. Model Building

**Figure 1:**
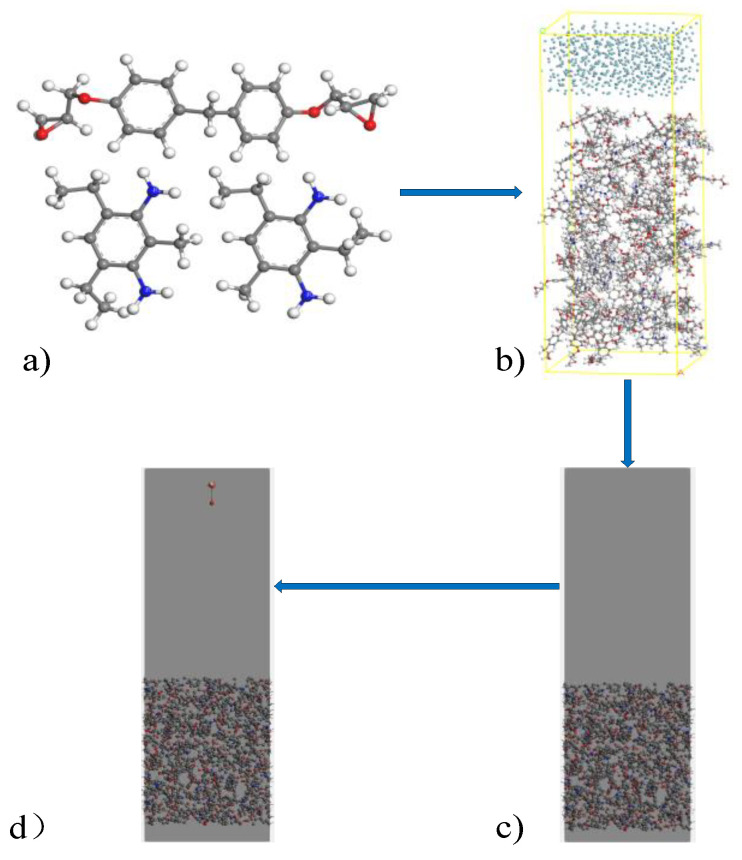
Construction process of cross-linked epoxy resin simulation model. (**a**) Molecular structure of bisphenol F epoxy resin monomer and curing agent monomer; (**b**) Constructing epoxy resin cross-linking interface model; (**c**) Import ReaxFF software; (**d**) Arrange the position and angle of incidence of high-energy particles.

In this paper, bisphenol F epoxy resin matrix and diethyltoluene diamine (DETDA) curing agent are used as the simulation research object to build the model. Figure 1 is the flow chart for establishing the cross-linked epoxy resin interface model. According to the steps in Figure 1, the periodic cross-linked epoxy resin interface model on the XOY plane is built, and the impact of high-energy particles on the surface model is simulated. According to the characteristics of ReaxFF force field, when the total number of atoms in the model reaches about 4000, stable and accurate simulation results can be obtained while guaranteeing calculation efficiency. Therefore, an amorphous mixture model with 60 DGEBF and 30 DETDA molecules and a density of 0.6 g/cm^3^ is established in this paper. Before the establishment of the cross-linked epoxy resin model, the periodicity of the model in the *Z*-axis direction is blocked by adding a vacuum layer of 15 Å, in which the argon molecular layer is filled. The cross-linking process of the model in the Z-direction is isolated to establish the periodic interface model on the XOY plane and then cross-link the amorphous model [24]. After the cross-linking procedure is completed, the argon molecular layer on the surface is deleted, and a surface model of cross-linked epoxy resin with 95% degree of cross-linking is obtained. The obtained model is imported into AMS software and a density of 1.19 g/cm^3^ model is obtained by 100 ps relaxation at 298 K and 0.1013 MPa using NPT system [24]. The addition of a vacuum layer in the *Z*-axis of the cell provides space for the motion of energetic particles, and the final cell volume is 30.195793 Å × 30.195793 Å × 100.00 Å. The position of high-energy particles is set on a plane at the top of the *Z*-axis of the cell. The horizontal position of each high-energy particle is randomly distributed, and the incident direction is vertical to the XOY plane.

### 2.2. Simulation Settings

In order to simulate the dynamic and thermodynamic effects of high-energy particles bombarding the insulation interface, a micro-canonical ensemble (NVE) is selected for the simulation system, in which the atomic number N, volume V and energy E remain unchanged. When high-energy particles with certain kinetic energy are incident, the energy E of the system rises, while the form and parameters of potential in NVE ensemble are fixed, and the position of the particles is determined by the motion of the particles themselves. The energy of the system will change the temperature of the system. Therefore, the use of NVE ensemble can simultaneously reflect the kinetic and thermodynamic effects caused by high-energy electric particle bombardment.

The initial temperature of the simulation system is 298 K, and the air pressure is set to 0.1013 MPa. Due to the high kinetic energy of high-energy particles in the simulation process, it is necessary to set a short simulation step in order to make the simulation converge. The simulation step size is 0.05 fs, the total number of simulation steps is 2.0 × 10^6^ steps, and the total time is 100 ps. The four energetic particles *H*_3_*O^+^*, *NO^+^*, *OH^−^* and *O*_3_*^−^* carry energy of 1, 4, 7 and 10 eV in different combinations and are incident along the epoxy resin interface in the negative direction of *Z*-axis at intervals of 1 ps. It takes 100 ps to simulate the accumulation effect of high-energy particle impact under pulsed partial discharge.

## 3. Simulation Results and Analysis

### 3.1. Analysis of Small Molecular Products of Epoxy Resin Impacted by High-Energy Particles

**Figure 2:**
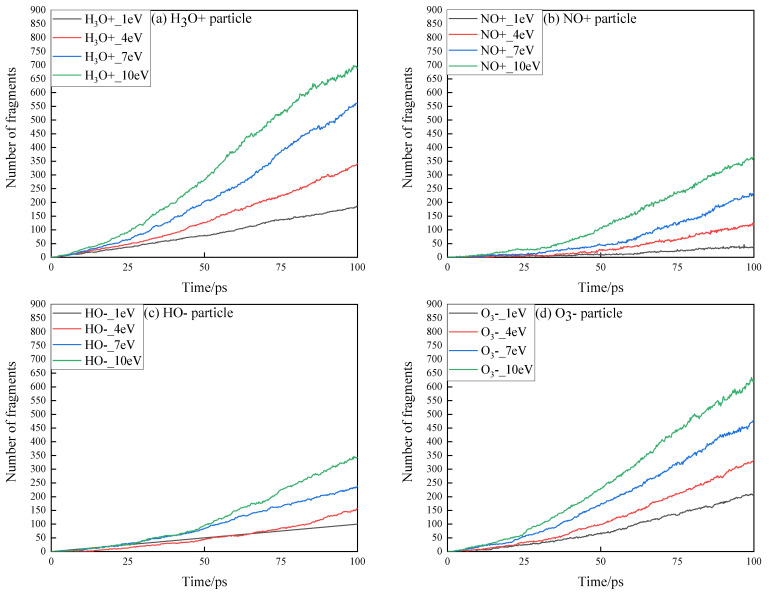
Variation trend of fragment number caused by impact of four high-energy particles on epoxy resin interface with different energy. (**a**) Changes in the number of *H_3_O^+^* particle fragments with different energies; (**b**) Changes in the number of *NO^+^* ion fragments with different energies; (**c**) Changes in the number of *HO^−^* ion fragments with different energies; (**d**) Changes in the number of *O_3_^−^* ion fragments with different energies.

In the simulation of high-energy particle impact, all atoms in the epoxy resin surface model are cross-linked and can be regarded as one molecule. Therefore, the total number of molecules in a cell can represent the volume of debris and damage caused by impact of energetic particles. The volume of debris produced by four high-energy particles impacting the surface of insulating material with different energy tends to change, as shown in Figure 2.

First, as the energy carried increases, the volume of debris produced by the impact of various high-energy particles increases. Among them, *H*_3_*O^+^* and *O*_3_*^−^* are the strongest ones to destroy the surface of epoxy resin. At the same time, the increase in the volume of debris produced is not linear with the increase in the energy of high-energy particles. When all types of high-energy particles carry more or less energy, there is little difference in the volume of debris generated in the first half of the simulation process, while the speed of debris generated by the impact of high-energy particles with higher energy in the second half is greatly increased. This is mainly due to the fact that the kinetic energy carried by high-energy particles when impacted on the surface of insulating material changes into thermal energy, which makes the cell temperature rise sharply.

**Figure 3:**
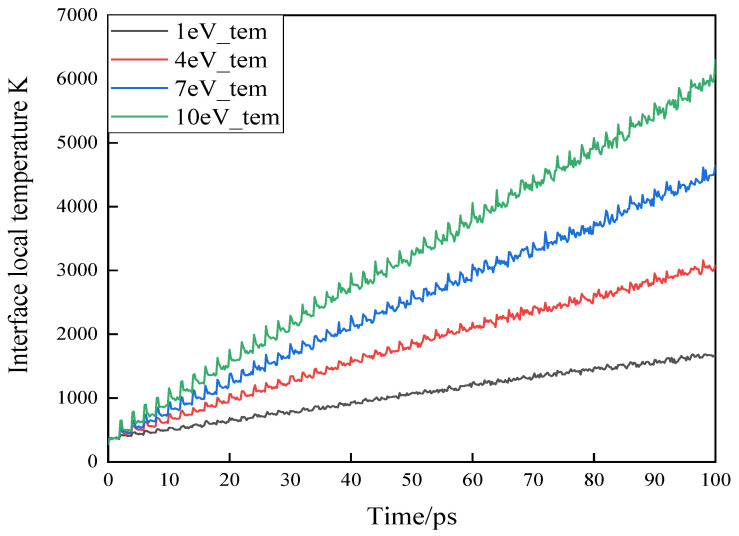
Local temperature change trend of epoxy resin.

The change trend of local temperature at the interface of epoxy resin impacted by high-energy particles with different energy is shown in Figure 3. During the impact process of *H*_3_*O^+^* carrying different energies, the temperature in the cell will rise to over 4000 K, which will cause the temperature in the local area of the epoxy material surface to rise sharply. When the energy of high-energy particles is low, most of the incident particles cannot directly destroy the surface of the epoxy resin, usually only because the high-energy particles have their own chemical reaction activity to attack the epoxy resin. The collision between high-energy particles and the surface of epoxy resin results in heat energy which causes local temperature rise, while the thermal conductivity of epoxy resin is poor, at only 0.2~2.2 W/mK. Local temperature rise caused by particle impact cannot diffuse in time, which leads to rapid accumulation of local heat and temperature rise, making epoxy resin subject to more severe erosion.

The simulation results of main small molecular products generated by the impact of high-energy particles with various parameters are analyzed to study the aging process of small molecular products and insulating materials analyzed in partial discharge experiments. Epoxy resin is an organic insulating material, and the damage of carbon skeleton structure is directly related to the formation of carbon-containing, small molecular products. It can be found that when *NO^+^* and *HO^−^* particles interact, the amount of C2 and C3 products in the small molecule products produced is very low, so the mass loss under the corresponding conditions is smaller. In the impact simulation of *H*_3_*O^+^*, the small molecular products of C1 and C2 produced by *H*_3_*O^+^* are significantly higher than those of other particles. After 40 ps, as the temperature of the simulation system increases, the epoxy resin molecules are more likely to be damaged by particle impact, and the rate of small molecule products production in each system increases significantly. In addition to carbon-containing, small molecular products, the main small molecular products under the action of particles are *H_2_O*, *H_2_*, *CO_2_*, etc. If there are few *H*_3_*O^+^* and *O*_3_*^−^* particles in the environment where partial discharge occurs, it is difficult to cause significant damage to the surface of epoxy resin when there are only a few *NO^+^* and *HO^−^* particles. *H*_3_*O^+^* will be released, resulting in a large amount of hydrogen gas. Because of its strong oxidation, *O*_3_*^−^* particles will further corrode the epoxy resin while breaking the molecular structure of the epoxy resin under impact.

### 3.2. Surface Damage and Mass Loss Characteristics of Epoxy Resin

**Figure 4:**
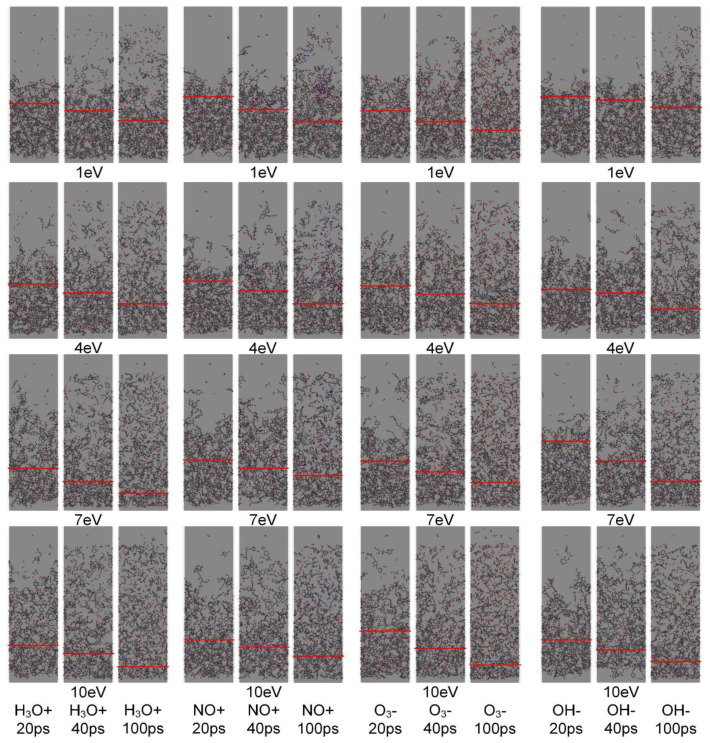
Structural change of interface model under the action of high-energy particles.

The interface structure change of epoxy resin under the action of high-energy particles is shown in Figure 4. The analysis of model structure changes during simulation shows that with the development of simulation process, the small molecular debris generated on the surface of epoxy resin diffuses in the empty area along the *Z*-axis when the energy of high-energy particles is low. However, most of the small molecular debris are still concentrated in the lower half, and the average molecular mass of the small molecular debris is higher than that of high-energy particles. At the same time, when the energy carried by the high-energy particles is low, during the impact process of the high-energy particles, most of the high-energy particles rebound after striking the surface of the epoxy resin without reacting with the epoxy resin or generating small molecular debris.

When the energy of high-energy particles is high, obvious damage occurs on the epoxy resin surface at an earlier stage. The high-energy particles with 7 eV already caused relatively long and deeper void damage at an earlier stage compared to the low-energy particles. In addition, most of the high-energy particles can impact on the surface of epoxy resin to produce small molecular debris, and the average incident depth is significantly increased.

**Figure 5:**
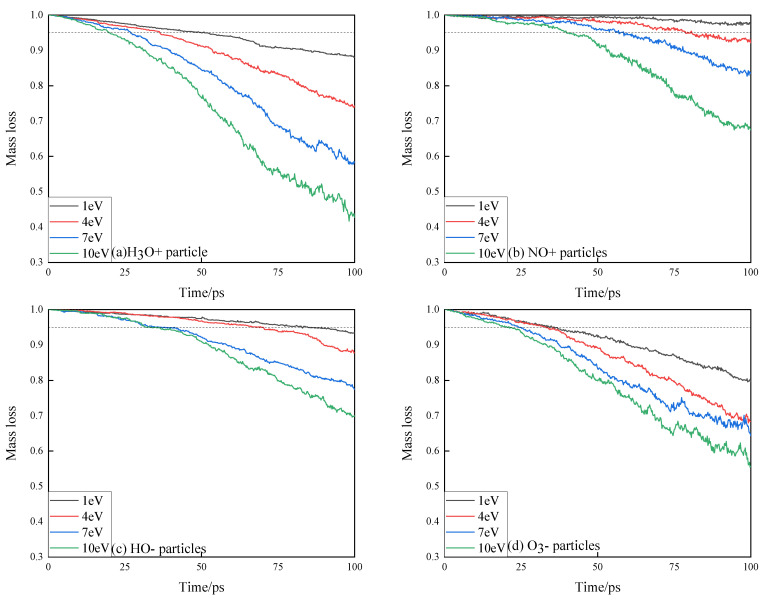
Normalized mass loss curve of epoxy resin under the action of high-energy particles. (**a**) Normalized mass loss curve of epoxy resin under the action of *H_3_O^+^* particles; (**b**) Normalized mass loss curve of epoxy resin under the action of *NO^+^* particles; (**c**) Normalized mass loss curve of epoxy resin under the action of *HO^−^* particles; (**d**) Normalized mass loss curve of epoxy resin under the action of *O_3_^−^* particles.

Normalized mass statistics of epoxy resin under high-energy particle impact during simulation are shown in Figure 5. The trend of epoxy resin variation can indicate the degree of erosion of epoxy resin under impact of high-energy particles. According to GBT 20112-2006 (the evaluation and identification of electrical insulation structure), when the quality loss of insulation material reaches 5%, the insulation material can be considered to be ineffective [25]. It can be seen that the mass loss of epoxy resin material is small when the energy of *NO^+^* and *HO^−^* particles is low, and the rate of mass loss is small during the simulation process of the first 50 ps or so. In combination with the side-cut snapshot of the simulation model in Figure 4, it can be found that although the impact action of high-energy particles will cause the cross-linkage structure of epoxy resin to react or break with high-energy particles, the amount of small molecular products that completely break off the surface of epoxy resin is small. The mass loss of epoxy resin under the action of *H*_3_*O^+^* and *O*_3_*^−^* particles is significantly higher than that under the other two conditions. At the end of the simulation process, the mass loss of the model under *H_3_O^+^* is higher than that under the same condition of *O_3_^−^* particles. However, *O*_3_*^−^* is easy to produce during impact, and its strong oxidation will also cause corrosion to the epoxy resin, which leads to the mass loss of epoxy resin under the action of *O*_3_*^−^* particles reaching 5% earlier, making the insulating material invalid earlier.

### 3.3. The Main Small Molecule Products of Epoxy Resin Surface Damage

It is very difficult to analyze the small molecular products produced by the impact of high-energy particles of partial discharge in experiments, and the generation of these small molecular products is directly related to the aging of insulating materials. Therefore, the generation and mechanism of small molecular products caused by the impact of various high-energy particles on epoxy resin are analyzed.

Epoxy resin is an organic insulating material, and the damage of carbon skeleton structure is directly related to the formation of carbon-containing, small molecular products. It can be found that when *NO^+^* and *HO^−^* particles interact, the amount of C2 and C3 products in the small molecule products produced is very low, so the mass loss under the corresponding conditions is smaller. In the impact simulation of *H*_3_*O^+^*, the amount of small molecular products of C1 and C2 produced by *H*_3_*O^+^* is significantly higher than that of other high-energy particles. After 40 ps, with the increase in the temperature of the simulation system, epoxy resin molecules are more likely to be damaged by high-energy particle impact, and the production rate of small molecular products in each system increases significantly. In addition to carbon-containing, small molecular products, the main small molecular products under the action of high-energy particles are *H_2_O*, *H_2_*, *CO_2_*, etc.

Combined with the mass loss information in Figure 5, it can be found that if the *H_3_O^+^* and *O_3_^−^* particles produced in the environment where partial discharge occurs are small, it is difficult to cause significant damage to the surface of epoxy resin when there are only a few *NO^+^* and *HO^−^* particles. *H*_3_*O^+^* will release hydrogen ions after impact contact with epoxy resin, resulting in a large amount of hydrogen gas. Because of its strong oxidation, *O*_3_*^−^* particles will further corrode the epoxy resin while breaking the molecular structure of the epoxy resin under impact.

### 3.4. Polar Effect of Surface Damage of Epoxy Resin

**Figure 6:**
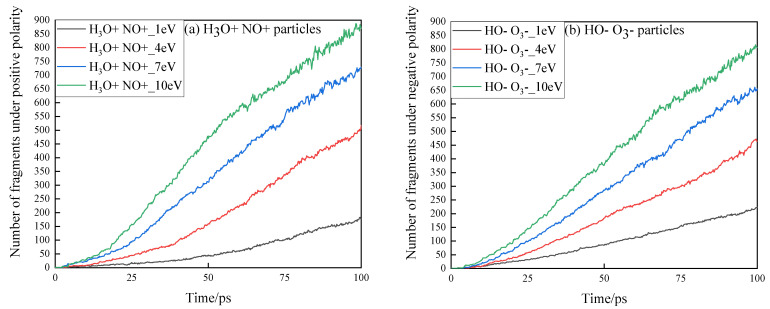
Variation trend of small molecular fragment products of epoxy resin under the action of positive and negative high-energy particles. (**a**) Variation trend of small molecular fragment products of epoxy resin under the action of positive high-energy particles; (**b**) Variation trend of small molecular fragment products of epoxy resin under the action of negative high-energy particles.

**Figure 7:**
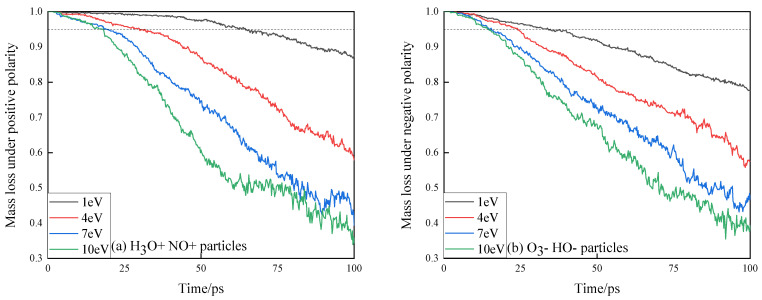
Normalized mass loss curve of epoxy resin under the action of positive and negative high-energy particles. (**a**) Normalized mass loss curve of epoxy resin under the action of positive high-energy particles; (**b**) Normalized mass loss curve of epoxy resin under the action of positive and negative high-energy particles.

In DC discharge experiments, the positive and negative discharge polarities often have effects on the starting discharge voltage, flashover voltage, damage to insulating materials, etc. Therefore, this paper presents a comparative study on the change trend of the number and mass loss of small molecular fragment products of epoxy resin under the combined impact of positive and negative high-energy particles, as shown in Figure 6 and Figure 7.

In Figure 6, under various energy conditions carried by high-energy particles, the mass loss of epoxy resin under the action of negative high-energy particles increases faster than that under the action of positive high-energy particles. At 14.4 ps, the mass loss of the epoxy resin model subjected to the impact of 10 eV high-energy particles reaches 5%, which is higher than that of the epoxy resin model subjected to the simultaneous action of single positive-polar high-energy particles. Under the same energy condition, the mass loss of epoxy resin reaches 5% at 18 ps under the action of positive energetic particles. When the energy carried by the high-energy particles is low, the difference between the positive and negative high-energy particles on the erosion of epoxy resin is greater. When the energy carried by high-energy particles is 1 eV, the time for the mass loss to reach 5% under the action of positive and negative high-energy particles is 64.4 ps and 34 ps, respectively. When the energy of charged particles is 4 eV, it is 29.8 ps and 24 ps, respectively. This shows that the working life of epoxy resin is longer under the action of positive energetic particles.

**Figure 8:**
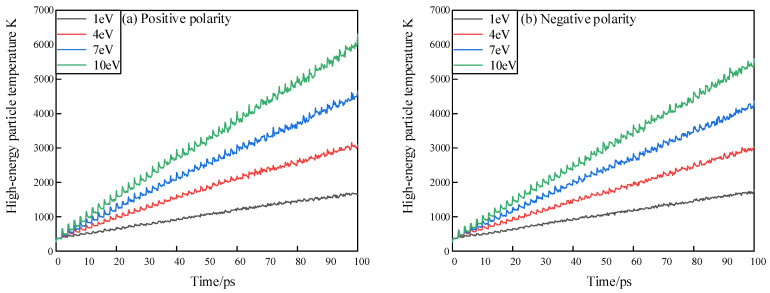
Temperature variation curve of simulation system under the action of positive and negative high-energy particles. (**a**) Temperature variation curve of simulation system under the action of positive high-energy particles; (**b**) Temperature variation curve of simulation system under the action of negative high-energy particles.

With the development of impact, the temperature of the simulation system gradually increases. The local temperature rise of epoxy resin interface under the impact of positive and negative high-energy particles is limited, as shown in Figure 8, and the temperature under the action of high-energy particles with negative polarity is always higher than that under positive polarity. This leads to the impact of positive energetic particles at the same time, which will locally raise the temperature of the epoxy resin surface to a higher temperature and aggravate the erosion. At the same time, the unstable *O*_3_*^−^* and *HO^−^* in the negative particles have strong oxidation after collision with the epoxy resin, which makes the cross-linking structure of the epoxy resin more fragile and the surface of the epoxy resin more vulnerable to erosion.

## 4. Conclusions

The effect of high-energy particle impact on the insulation degradation of epoxy resin is analyzed by ReaxFF simulation force field. The decomposition process, mechanism and characteristics of insulating materials were analyzed at the micro atomic level, and the following conclusions are drawn:

(1) The high-energy particles produced by partial discharge will cause irreversible erosion on the surface of epoxy resin material. The simulation results show that *O*_3_*^−^* particles have the strongest erosion effect on the surface of epoxy resin material under the action of high-energy particles alone. High-energy particles not only impact the surface of epoxy resin and interrupt its chemical structure, but also convert its kinetic energy into potential energy, resulting in a sharp rise in the system temperature and the cracking of epoxy resin. This also shows that when the insulating material is subjected to partial discharge, the local temperature will rise sharply, resulting in insulation deterioration.

(2) In the simulation of various high-energy particles carrying different sizes of energy, the erosion effect on epoxy resin is characterized by mass loss. It can be found that compared with charged particles with higher energy, the erosion effect of charged particles with lower energy on epoxy resin materials is less in the initial stage, but it is very obvious in the later stage of impact development and accumulation.

(3) In the study of the positive and negative polarity of high-energy particles, it is found that the corrosion effect of negative charged particles on epoxy resin is more obvious. This is because the temperature rise of the system increases under the impact of negative charged particles, and the negative particles *O*_3_*^−^* and *HO^−^* are unstable and have strong oxidation, which makes the cross-linking structure of epoxy resin more fragile.

## Data Availability

All data are available in the main text.
